# Effects of Al Element on the Microstructure and Properties of Sn-9Zn Solder Alloy

**DOI:** 10.3390/ma19061159

**Published:** 2026-03-16

**Authors:** Jiaojiao Yang, Yuanqi You, Gaohua Jiang, Caiju Li

**Affiliations:** 1 Faculty of Resources and Environmental Engineering, Yunnan Vocational Institute of Energy Technology, Qujing 655001, China; yjj961127@163.com; 2 Faculty of Materials Science and Engineering, Kunming University of Science and Technology, Kunming 650093, China; yyqkust@126.com (Y.Y.); lcj@kust.edu.cn (C.L.)

**Keywords:** Sn-9Zn alloy, lead-free solder, microalloying, mechanical property, corrosion resistance

## Abstract

The application of microalloying technology has significantly improved the mechanical properties, oxidation resistance, and corrosion resistance of the Sn-9Zn-xAl-series solder. The effects of Al addition on microstructural evolution and service-related performance of the solders were systematically investigated using a combination of characterization techniques, including scanning electron microscopy/energy-dispersive X-ray spectroscopy (SEM/EDX), differential scanning calorimetry (DSC), tensile testing, spreading testing, thermogravimetry (TG), and potentiodynamic polarization measurements. Microstructural characterization reveals that an optimal content of Al reacts with the Sn-Zn matrix to form AlZnSn intermetallic compounds (IMCs), which effectively refines the Zn-rich precipitates and eutectic lamellar structure. Concomitantly, the formation of second-phase strengthening contributes to a significant enhancement in the tensile strength of the solder alloys. Specifically, the Sn-9Zn-0.8Al solder exhibits a tensile strength of 87 MPa, corresponding to a 37% increment compared to the base Sn-9Zn alloy, whereas the elongation is reduced to 14.1%. Moreover, the in situ-formed Al_2_O_3_ passive film provides effective protection for the solder matrix, inhibiting oxidation induced by oxygen atoms and corrosion caused by chlorine ions, thereby remarkably improving the oxidation and corrosion resistance of the alloy. Collectively, these findings demonstrate that Al microalloying can substantially enhance the strength, oxidation resistance, and corrosion resistance of Sn-9Zn solder; however, a trade-off between wettability and ductility needs to be carefully considered for practical applications.

## 1. Introduction

Sn-Pb solder is the most widely used material for brazing connections due to its excellent welding performance and reasonable price [1,2,3]. However, lead (Pb) and its compounds are classified as toxic and hazardous substances. Excessive exposure may cause adverse effects on human health and ecological environments [1,4]. In addition, tin-lead solder also has shortcomings such as mismatch of thermal expansion coefficient, solder pad plating, unstable alloy structure, and poor creep resistance, which can no longer satisfy the reliability requirements of electronic products in the new era, so research and development are urgently needed for a new type of solder alloy with excellent performance and controllable cost [5,6].

Compared with Sn-Cu [7], Sn-Ag [8], Sn-Bi [9,10], and Sn-In [10] system solders, the melting point of the Sn-Zn system is the closest to the traditional Sn-Pb solder, and the lowest cost, the soldering process, and equipment are closest to Sn-Pb solder, which is used in the soldering process of most equipment. Sn-9Zn solder has higher strength than Sn-3.5Ag solder and Sn-37Pb solder [1,8,11]. As an element with abundant reserves and low toxicity, Zn renders Sn-Zn alloys as potentially lead-free solder alternatives. Sn-Zn-based lead-free solders exhibit considerable potential in terms of cost-effectiveness and environmental friendliness. Existing studies have demonstrated that an appropriate addition of Zn can refine certain intermetallic compounds (IMCs) and induce a dispersion strengthening effect; however, excessive Zn may exert an adverse impact on microstructural evolution [12]. So, Sn-9Zn solder is considered a lead-free solder alternative to traditional solder.

Some Sn-9Zn-based solders have been used globally, such as Sn-9Zn-3Bi and Sn-9Zn-0.2Cr, but due to their insufficient wettability and oxidation resistance, their wide application is limited [13,14,15,16]. The poor oxidation resistance of Sn-9Zn is mainly due to the active nature of the Zn element, which makes the solder easily oxidized and affects the service reliability of the solder. In, Bi, Ga, and Al can significantly reduce the melting point of Sn alloys [2,17]. At the same time, Al can reduce the alloy’s surface tension [18]. Specifically, Al exhibits a stronger affinity for O. At elevated temperatures, Al tends to undergo preferential oxidation and forms a dense Al_2_O_3_ film on the surface. This film acts as a diffusion barrier to reduce the effective diffusion flux of O, thereby significantly retarding the subsequent oxidation process [19]. Adding an appropriate amount of Al can improve the high-temperature stability of the solder. Since the Al is adsorbed on the interface’s grain boundary, the intermetallic compound’s grain size can be reduced, and the mechanical properties of the solder can be improved [20].

Lead-free solder has established itself as an indispensable material in the field of electronics manufacturing. In this paper, Sn-9Zn alloy solders with great development prospects are selected, and Sn-9Zn solder alloys with different contents of Al are investigated by the methods of microalloying, mechanical properties, oxidation resistance, and corrosion resistance. Finally, a new Sn-Zn-Al solder alloy was developed to enrich the theoretical system of Sn-based alloy solder.

## 2. Experimental Methods

According to Sn-9Zn-xAl (x = 0, 0.2, 0.4, 0.6, 0.8 wt.%), 3 metal particles of Sn, Zn, and Al were weighed, and their metal purity was greater than 99.99%. After the sample composition was prepared, the tubes were sealed using a hydrogen-oxygen torch sealing machine under a vacuum of 10^−4^ Pa. The sealed samples were then placed in a 16-channel swing melting furnace for smelting. The furnace temperature was raised from room temperature to 700 °C and held for 2 h to ensure complete melting and thorough reaction of the alloy, with the furnace swinging at a rate of 6 r/min. Subsequently, the furnace temperature was cooled to 300 °C at a rate of 10 °C/min and maintained for 30 min. To suppress the formation of coarse Zn-rich phases in the microstructure, the samples were immediately quenched in 50 °C water after removal. The specifications of the experimental raw materials and equipment are summarized in Table 1 and Table 2, respectively.

The cooled samples were subjected to Inductive Coupled Plasma Emission Spectrometer (ICP) detection, and the results are shown in Table 3. The Sn-9Zn-xAl alloy was cut into a size of φ15 × 10 mm, polished with SiC sandpaper to 3000#, and polished using a polishing cloth with diamond paste. The alloy surface was then etched using a solution of 4 vol% nitric acid (HNO_3_) in ethanol (C_2_H_5_OH). The microstructure was observed by SEM. After observing the morphology, the grinding and polishing processes were repeated. The samples were connected with copper wires using epoxy resin to expose the surface of the working electrode, and the potentiodynamic polarization test was performed using a CHI760E electrochemical workstation under the condition of 3.5 wt.% NaCl electrolyte [1].

**Figure 1:**
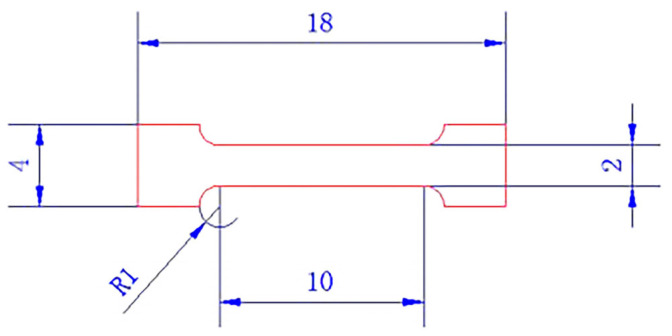
Schematic of the tensile specimen dimensions.

The melting properties of the alloys were determined using differential scanning calorimetry (DSC). The solder sample was 7 ± 0.05 mg, and the experimental reference plate was an Al plate. The whole experiment was protected by high-purity argon gas, and the argon gas flow was 50 mL/min. Staged heating was applied, heating from 140 °C to 250 °C at a rate of 10 °C/min, and cooling to room temperature at a rate of 5 °C/min. The tensile test was carried out according to the national standard GB/T 228.1-2021 [21,22]. The smelted sample was processed by an electric spark-cutting machine according to the specified size. A schematic diagram of the tensile specimen dimensions is shown in Figure 1. The experiments were carried out at room temperature with a stretching rate of 0.6 mm/min. Three parallel experiments were performed for each solder alloy.

The oxidation resistance of the alloys was characterized by thermogravimetric analysis (TGA). The thermogravimetric analyzer of NETZSCH was used in the experiment; the sensitivity was less than 0.1 μg, and the experimental atmosphere was oxygen. During the experiment, a sample of about 10 mg was used, the temperature was raised to 250 °C at a rate of 10 °C/min, the temperature was kept for 160 min, and the crucible material used was Al_2_O_3_.

## 3. Results and Discussion

### 3.1. Microstructural Characteristics

**Figure 2:**
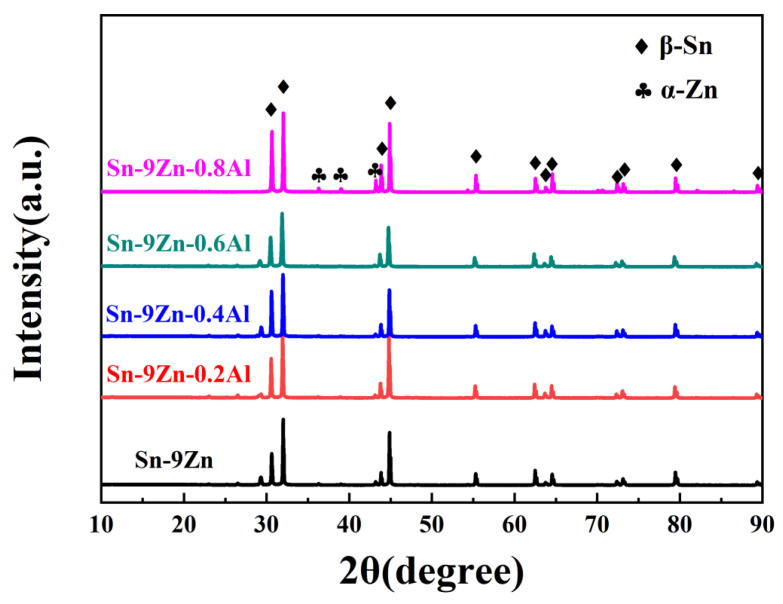
X-ray diffraction patterns of Sn-9Zn-xAl solder alloy.

X-ray diffraction patterns of Sn-9Zn-xAl solder alloy are illustrated in Figure 2. There is no diffraction peak of aluminium in all samples. All samples have only the presence of β-Sn and α-Zn phases in the X-ray diffraction spectrum. From the Sn-Zn binary phase diagram [23], the Sn-Zn alloy is composed of β-Sn and α-Zn phases, and there is no intermetallic compound between Sn and Zn. Due to the low solid solubility of Zn in Sn (about 0.4 wt.% at 180 °C), the Zn phase will separate.

**Figure 3:**
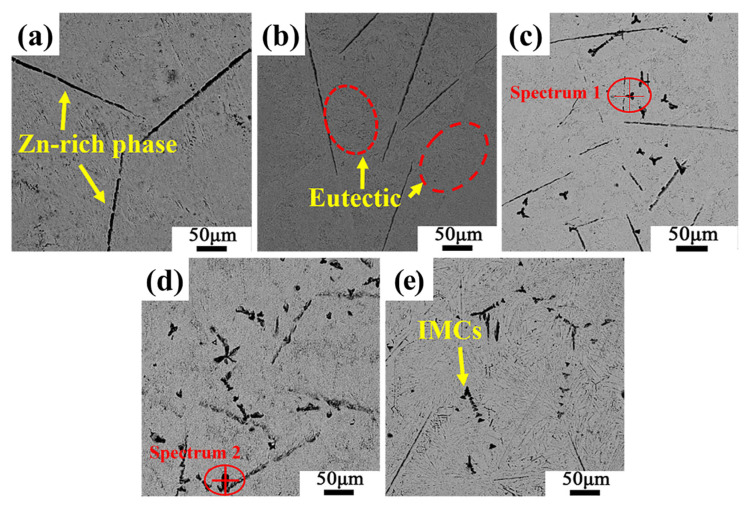
Microstructure of Sn-9Zn-xAl solder alloy: (**a**) x = 0; (**b**) x = 0.2; (**c**) x = 0.4; (**d**) x = 0.6; (**e**) x = 0.8.

Figure 3 is the SEM morphology picture of Sn-9Zn-xAl solder alloy. It can be seen from Figure 3a that Sn-9Zn is composed of a black primary α-Zn-rich phase, eutectic Sn-9Zn, and a grey β-Sn matrix. Due to non-equilibrium solidification, the rod-like primary α-Zn phase is randomly distributed in the β-Sn matrix, and the fine needle-like Zn phase and Sn are intertwined to form a eutectic structure. The coarse rod-shaped primary Zn phase is detrimental to the mechanical properties of the material. During the plastic deformation process, the coarse rod-shaped primary Zn phase hinders the movement of dislocations, causing dislocations to accumulate near the α-Zn phase and causing stress concentration, which leads to cracks in the α-Zn phase, originated and expanded here [24]. As shown in Figure 3c, when the additional amount of Al reaches 0.4 wt.%, the microstructure changes, and irregular new petal-like second-phase microstructures appear in the typical eutectic layer. And with the increase of Al content, the new dark-grey phases in the matrix increased and tended to be finely aggregated, and the phases were mostly triangular or quadrangular. In addition, the coarse Zn-rich phase also becomes significantly finer, and it can be seen that the addition of an appropriate amount of Al refines the structure of the second phase.

**Figure 4:**
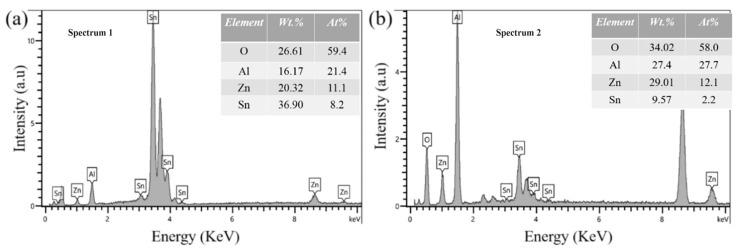
The spot scan results of position: (**a**) Figure 3c, (**b**) Figure 3d.

The composition of the intermetallic compound was analyzed by EDX. Figure 4 shows the point scan results for Position 1 in Figure 3c, and Position 2 in Figure 3d. This phase is an Al-containing or Al-rich phase, which may consist of aluminium-rich oxides (e.g., Al_2_O_3_), given the high activity of Al and its strong tendency to oxidize. The oxygen signal may originate from oxidation of the particle itself, sample preparation, or a surface oxide layer formed after polishing. Meanwhile, the scanning results indicate that the atomic ratio of Al to Zn is close to 0.5, suggesting the possible presence of an Al_2_Zn intermetallic compound. The presence of this compound was also found in previous studies [25,26].

It can be seen that after adding elemental Al to Sn-9Zn alloy, the α-Zn phase in the alloy is reduced and transformed into a finer structure. In addition, Zn is very active and can form compounds with Al, so the coarser Zn-rich eutectic layer in Sn-9Zn-xAl alloy disappears, forming fine eutectic clusters and dispersing Al_2_Zn intermetallic compounds. When the addition amount of Al is less than 0.4 wt.%, no regular dark-grey phase is observed. It is speculated that there are two reasons. On the one hand, because the amount of Al added at this time is relatively small, Sn is dominant, and the Al atoms cannot compete with the Sn atoms in the eutectic alloy to form compounds with Zn. On the other hand, Al_2_Zn intermetallic compounds have lower Gibbs formation energy and lower growth activation energy from a thermodynamic point of view, so the appearance of compounds cannot be observed at this time [27].

### 3.2. Melting Properties

**Figure 5:**
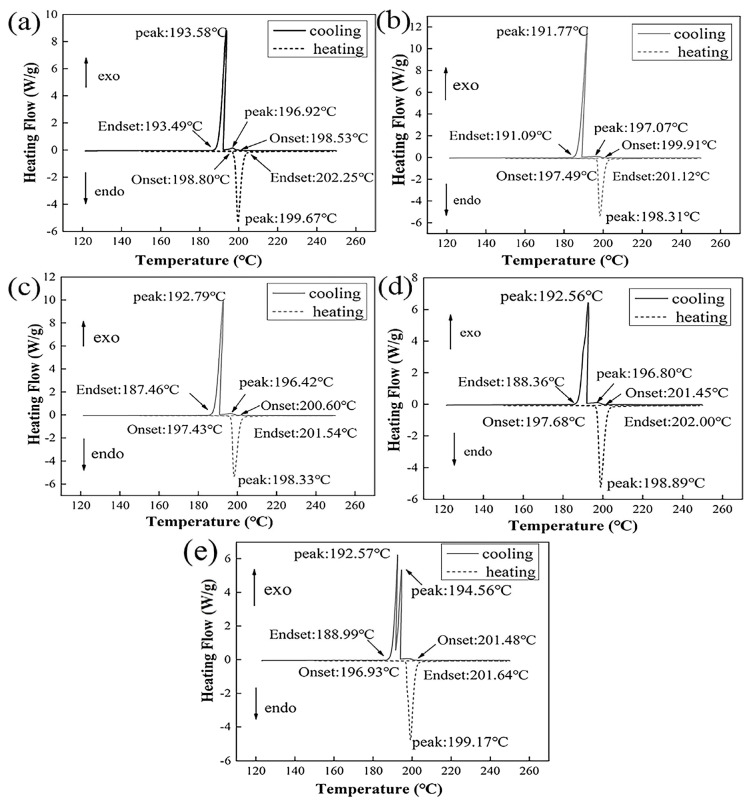
DSC curves of Sn−Zn−xAl solder alloys: (**a**) x = 0; (**b**) x = 0.2; (**c**) x = 0.4; (**d**) x = 0.6; (**e**) x = 0.8.

The DSC curves of the heating and cooling of Sn-9Zn-Al and the data results of specific measurements are shown in Figure 5 and Table 4, respectively. It can be seen from Figure 5 that the melting point of Sn-9Zn-xAl solder alloy has a tendency to first decrease and then increase with the addition of Al, and the melting point increases slightly with the increase of Al. Compared with Sn-9Zn (199.09 °C), the addition of the Al makes the melting point of the solder alloy slightly lower [28].

Two endothermic peaks appeared in both Sn-9Zn-xAl solder alloys. With the increase of the Al, the positions of the two peaks became more and more obvious and the distance between the peaks became larger. The two peaks appearing in the exothermic DSC curve correspond to the eutectic structure and the formed intermetallic compound, respectively.

In Table 4, with the increase of Al content, the *T_peak_* value of the first peak only changes slightly; its value is about 1 °C, and the value of the second peak changes about 1~3 °C. It can be seen that the content of Al has a significant effect on *T_peak_*. The value has little effect. It can be seen from the table that the temperature at that specific content of Al affecting the solidus does not exceed 2 °C, and the effect on the temperature of the liquidus is about 1 °C. The melting range of Sn-9Zn-0.2Al is 1.56 °C lower than that of Sn-9Zn (5.19 °C). With the increase of Al content, the two exothermic peaks are also more and more obvious.

### 3.3. Mechanical Properties

**Figure 6:**
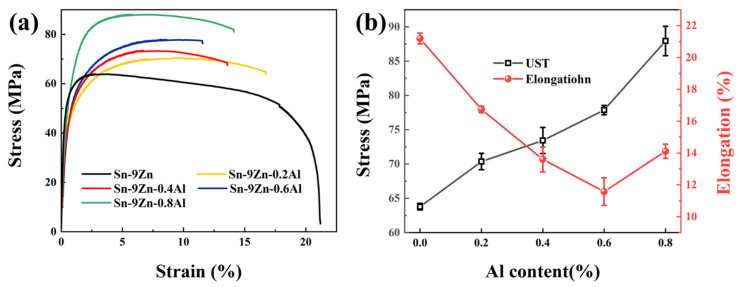
Tensile properties of Sn-9Zn-xAl solder alloys: (**a**) typical tensile stress–strain curves; (**b**) ultimate tensile strength (UTS) and elongation.

Figure 6 is the stress–strain curve of Sn-9Zn-xAl solder alloy. It can be seen from Figure 6b that the tensile strength of Sn-9Zn-xAl solder alloy increases with the increase of Al content, and the tensile strength of Sn-9Zn-0.8Al solder alloy is 87 Mpa. Compared with Sn-9Zn, Sn-37Pb, and Sn-3.0Ag-0.5Cu, there are increases of 70%, 64%, and 78.6%, respectively [11,29]. As for the elongation, the elongation decreased obviously after adding the Al compared with Sn-9Zn. However, when the Al content increases to 0.6 wt.%, the Sn-9Zn-0.6Al elongation reaches about 11%, which is decreased compared with other Sn-9Zn-xAl solder alloys.

**Figure 7:**
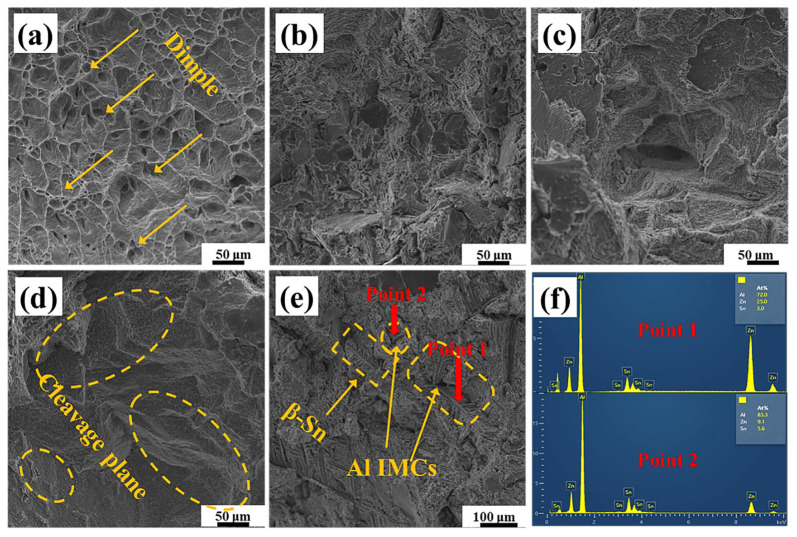
Tensile fracture morphology of Sn-9Zn-xAl alloy: (**a**) Sn-9Zn solder alloys; (**b**) Sn-9Zn-0.2Al solder alloys; (**c**) Sn-9Zn-0.4Al solder alloys; (**d**) Sn-9Zn-0.6Al solder alloys; (**e**) Sn-9Zn-0.8Al solder alloys; (**f**) the spot scan results of (**e**).

Figure 7 shows the SEM morphology of Sn-9Zn-xAl solder alloys with different content. It can be observed in Figure 7a that the Sn-9Zn alloy fracture is a typical ductile fracture. With the increase of Al content, the dimples disappeared. Small dimples were found only in 0.2 wt.%. It indicates that the material has undergone a transition from toughness to brittleness. With the increase of Al content in the matrix to 0.8 wt.%, dendritic intermetallic compounds can be seen in Figure 7e, and the specific energy spectrum information can be seen in Figure 7f. The element information of Point 1 and Point 2 is Al intermetallic compounds.

According to the Orowan-Ashby equation [30,31], as the Al is added into the Sn-9Zn alloy to generate the second-phase structure, the alloy strength increases. On the one hand, the second phase formed by Al is the largest region of mismatch at the grain boundary; dislocation barriers are generated near the grain boundary, and the accumulation of dislocations here increases the tensile strength. On the other hand, due to the movement restriction of dislocation density, it is difficult for the slip plane to find a suitable free movement direction, so the toughness decreases, which is manifested as a decrease in elongation. It can be found in Figure 3b that when a small amount of Al is added, the coarse zinc-rich phase in Sn-9Zn disappears and a fine acicular eutectic phase appears. The coarse Zn-rich phase easily becomes a place for crack initiation and propagation, which is not good for the plasticity of the solder alloy. However, with the increase of Al content, the IMCs appeared, so the elongation of the solder alloy increased slightly.

### 3.4. Antioxidant Properties

**Figure 8:**
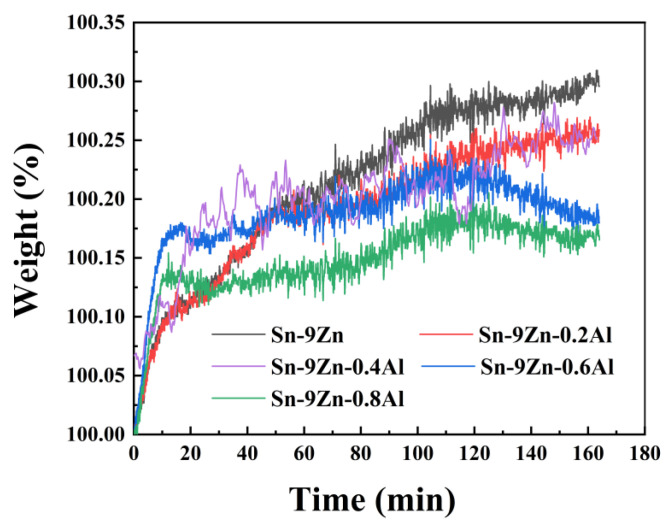
The thermogravimetric curve of Sn-9Zn-xAl alloy solders.

Figure 8 is the thermogravimetric curve of Sn-9Zn-xAl alloy. As can be seen from the figure, the Sn-9Zn-xAl solder alloy continued to increase in weight during the oxidation process. In the early stage of oxidation, the weight gain of the solder alloy is fast and then tends to be flat. With the increase of Al content, the weight gain ratio of the alloy decreases. The activity of the Al is higher than that of the Zn element, so when the self-diffusion of oxygen atoms contacts the solder, the Al that undergoes positive segregation preferentially reacts with oxygen to form a layer of Al_2_O_3_ monomolecular oxide film. The Al_2_O_3_ film is very dense and covers the molten metal. The solder surface forms a protective layer.

When the addition amount of aluminium element is lower than 0.4 wt.%, the weight gain ratio increases linearly. During the initial stage of oxidation, aluminum (Al) present on the surface of the molten solder first reacts with oxygen, thereby gradually forming a stable oxide film. The rapid oxidation of the alloy makes significant weight gain. At 160 min, the weight gain ratio was 0.30%. Due to the small amount of aluminium added at this time, the increased mass of the alloy was accompanied by a part of Zn oxides and Sn oxides. When the addition of aluminium is greater than 0.6 wt.%, the mass increases rapidly in the early stage of oxidation and during the heating process, and then becomes gentle. This is because the oxide film formation rate of Al_2_O_3_ is faster than the oxidation rate of Sn and Zn themselves. When the temperature continues to rise, it is difficult for the alloy to directly contact the oxygen in the air, and the oxidation rate of the solder is determined by the diffusion rate of the oxygen atoms on the surface of the solder in the Al_2_O_3_ single-molecule oxide film. In the initial stage of solder alloy oxidation, the surface reaction of the solder affects the oxidation resistance of the solder. However, with the increase of time and temperature, the Al_2_O_3_ oxide film thickens, and the diffusion rate of oxygen atoms becomes the controlling factor of the oxidation rate. The dense Al_2_O_3_ oxide film is beneficial to protect the solder and improving the oxidation resistance of the solder.

The mass weight gain percentage of the solder alloy in the early stage of Sn-9Zn oxidation is 0.32%; it can be seen that adding Al to the solder alloy can improve the oxidation resistance. When the addition of Al content reaches 0.8 wt.%, the minimum mass increase percentage of the solder alloy is about 0.17%, which is significantly improved compared with Sn-9Zn alloy.

### 3.5. Corrosion Resistance

**Figure 9:**
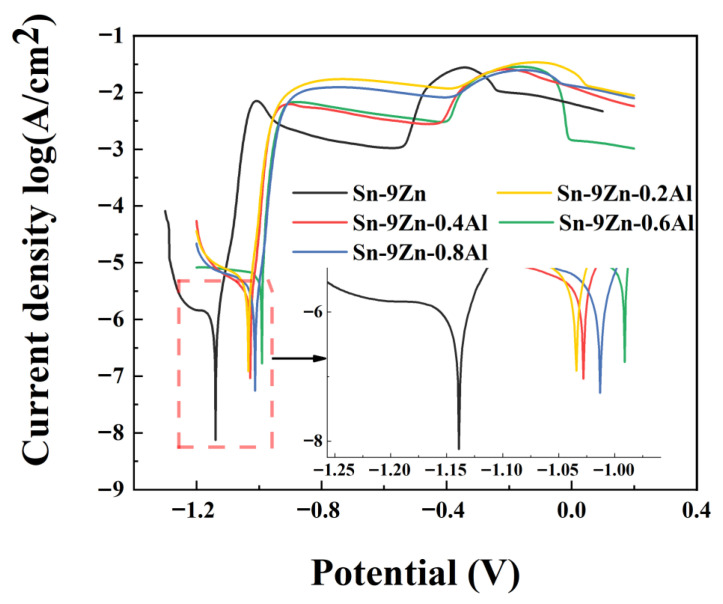
Potentiodynamic polarization curve of Sn-9Zn-xAl solder alloy in 3.5 wt.% NaCl solution.

Figure 9 shows the potentiodynamic polarization curve of Sn-9Zn-xAl solder alloy in 3.5 wt.% NaCl solution. It can be seen that the polarization curves of Sn-9Zn-xAl solder alloys show similar electrochemical characteristics. Due to the large difference between the standard electrode potentials of Sn and Zn (*E_Sn_* = −0.136 V, *E_Zn_* = −0.762 V), when the two contact each other, Sn is corroded as cathode and Zn as anode, forming galvanic corrosion [32]. Moreover, the difference in the content of Sn and Zn makes the area ratio of anode and cathode very large, which will cause serious local corrosion and pitting corrosion in the alloy, which is also the main reason for the poor corrosion resistance of Sn-9Zn solder [33,34].

As shown in Table 5, the corrosion potential of Sn-9Zn-xAl solder alloy shows a trend of first increasing and then decreasing. In general, with the addition of Al, the corrosion potential increases first and then decreases. The corrosion current density decreases first and then increases, and the passivation current density has the same trend. When the addition amount of Al is 0.2 wt.%, the corrosion potential is −1.036 V, which has been significantly improved compared with Sn-9Zn. When the addition amount of Al is 0.6 wt.%, the optimum corrosion potential is −0.972 V, which is improved compared with the original Sn-9Zn alloy and other Sn-9Zn-xAl alloys. Compared with the Sn-9Zn alloy, its corrosion current density and passivation current density have been improved to some extent, indicating that its corrosion resistance has been improved. It is mainly because the addition of Al refines the structure, makes the coarse-grain-rich Zn phase transition fine, and reduces the area and probability of pitting corrosion [35]. In addition, the addition of Al can form intermetallic compounds with Sn and Zn, and also deprives the elemental Zn and some eutectic alloys in the alloy matrix, thus reducing the active points of alloy corrosion, leading to improved corrosion resistance [36]. When the content of Al increases, its corrosion resistance is relatively strong, indicating that the intermetallic compounds formed in the structure also have a certain corrosion resistance [37].

## 4. Conclusions

In this work, the effects of Al addition on the microstructure, melting characteristics, mechanical properties, oxidation resistance, and corrosion resistance of Sn-9Zn alloy were studied in detail. The summary is as follows:

1. The melting range of Sn-9Zn-xAl alloy is slightly reduced by adding Al.
2. With increasing the amount of Al added to the Sn-9Zn alloy, a dense and fine butterfly-shaped intermetallic compound is formed, forming a second-phase strengthening. The addition of an appropriate amount of Al makes the coarse primary Zn-rich phase transition fine, which significantly improves the tensile strength of the alloy.
3. The thermogravimetric results indicate that Al microalloying significantly reduces the oxidation weight gain of Sn-9Zn. The weight gain for Sn-9Zn is approximately 0.58%, reaching a minimum of about 0.17% at x = 0.8 wt.%, which suggests that a dense Al_2_O_3_ film effectively inhibits subsequent oxygen diffusion and oxidation reactions.
4. In 3.5 wt.% NaCl solution, the corrosion potential shifts positively to approximately −0.992 V, and the corrosion current density decreases to about 15.39 μA/cm^2^ at x = 0.6 wt.%, demonstrating optimal corrosion resistance. This improvement is attributed to the refinement of Zn-rich phases, the weakening of micro-galvanic effects, and the consumption of part of the elemental Zn through the formation of intermetallic compounds (IMCs).

## Figures and Tables

**Table 1:** Experimental raw materials information sheet.

Element	Form	Size	Purity	Supplier
Sn	Granules	1–2 mm	99.99%	Zhongnuo Advanced Materials
Zn	Granules	3–5 mm	99.99%	Zhongnuo Advanced Materials
Al	Powder	25 μm	99.99%	Macklin

**Table 2:** Specifications of the experimental equipment.

Equipment	Model	Manufacturer	Key Parameters
16-Channel Melting Furnace	GSL-120_0X_-MGI-16	Hefei Kejing, Hefei, China	Maximum temperature: 1200 °C; Furnace body swing: ±15°
Hydrogen-Oxygen Flame Sealing Machine	OKFKJ1000	Walker Energy, Reno, NV, USA	Water consumption: 0.53 L/h; Vacuum level: ≤1 × 10^−6^ Pa; Flame temperature: ~3000 °C (adjustable)
Scanning Electron Microscope (SEM)	TESCAN VEGA 3	TESCAN, Brno, Czech Republic	Accelerating voltage: 10–30 kV; Magnification: <30,000×
Differential Scanning Calorimeter (DSC)	TA-DSC25	TA Instruments, New Castle, DE, USA	Sample mass: 5–10 mg; Max. temperature: <750 °C; Heating rate: 5 °C/min
Universal Testing Machine	AG-X Plus	Shimadzu, Kyoto, Japan	Ambient temperature; Tensile speed: 0.6 mm/min
Electrochemical Workstation	CHI760E	CH Instruments, Shanghai, China	Reference electrode: Saturated calomel electrode (SCE); Scan rate: 1 mV/s
Thermogravimetric Analyzer (TGA)	TG 209 F3	NETZSCH, Selb, Germany	Temperature range: 10–1000 °C; Heating rate: 0–200 K/min; Cooling time (1000 °C to ambient): <20 min
ICP-OES	5800	Agilent Technologies, Santa Clara, CA, USA	Wavelength range: 167–785 nm (full wavelength coverage); Optical resolution: ≤0.0065 nm

**Table 3:** Chemical composition of solder alloys (wt.%).

Specimen	Compositions (Unit: wt.%)				
	Zn	Al	Ag	Ni	Sn
Sn-9Zn	8.925	ND	ND	0.010	Bal.
Sn-9Zn-0.2Al	9.012	0.193	ND	0.016	Bal.
Sn-9Zn-0.4Al	8.769	0.4134	ND	0.009	Bal.
Sn-9Zn-0.6Al	9.185	0.606	ND	ND	Bal.
Sn-9Zn-0.8Al	8.673	0.895	0.001	ND	Bal.

Bal.: balance; ND: not detected.

**Table 4:** DSC heating data of Sn-9Zn-xAl (unit: °C).

Specimen	Heating		Pasty Range (*T_endset_*–*T_onset_*)	Cooling	Peak Temperature	
	*T_onset_*	*T_endset_*		*T_onset_*	1st	2nd
Sn-9Zn	196.57	201.76	5.19	198.53	193.5	196.9
Sn-9Zn-0.2Al	197.49	201.12	3.63	199.91	191.7	197.0
Sn-9Zn-0.4Al	197.43	201.54	4.11	200.60	192.7	196.4
Sn-9Zn-0.6Al	197.68	202.00	4.32	201.45	192.5	196.8
Sn-9Zn-0.8Al	196.93	201.64	4.71	201.48	192.5	194.5

**Table 5:** Electrochemical parameters for Sn-Zn-Al solder alloy in 3.5 wt.% NaCl solution.

Solder Alloys	OCP (V)	*E*_corr_ (V)	*i*_corr_ (μA/cm^2^)	*i*_pass_ (mA/cm^2^)
Sn-9Zn	−1.150	−1.132	59.59	31.53
Sn-9Zn-0.2Al	−1.045	−1.036	45.65	34.92
Sn-9Zn-0.4Al	−1.035	−1.028	15.39	13.56
Sn-9Zn-0.6Al	−0.980	−0.972	18.62	16.15
Sn-9Zn-0.8Al	−1.028	−1.021	16.58	14.07

OCP: Open circuit potential, *E*_corr_: self-corrosion potential, *i*_corr_: self-corrosion current density, *i*_pass_: passivation current density.

## Data Availability

The original contributions presented in this study are included in the article. Further inquiries can be directed to the corresponding author.
